# Effectiveness of topical gabapentin in the treatment of vulvodynia: a narrative synthesis

**DOI:** 10.3389/fpain.2023.1159268

**Published:** 2023-07-03

**Authors:** Mehmet Ergisi, Alexander Law, Nishant Chaudhari, Stefania Tsatsari, Kim Lawson, Christopher Jenner

**Affiliations:** ^1^ Imperial College London, London, United Kingdom; ^2^ Department of Biosciences and Chemistry, Sheffield Hallam University, Sheffield, United Kingdom; ^3^ Department of Biosciences and Chemistry, Imperical College London, London, United Kingdom, United Kingdom

**Keywords:** vulvodynia, dyspareunia, vulvar pain, gabapentin, anticonvulsants, pain management

## Abstract

Vulvodynia is a leading cause of dyspareunia in premenopausal women, causing considerable morbidity and sexual dysfunction. A multimodal approach is used to treat vulvodynia. Alongside psychosocial interventions and physiotherapy, pharmacological treatment such as oral gabapentin are used in the treatment of vulvodynia. Topical formulations of gabapentin have shown promise in animal models and case reports investigating its use in other pain conditions. The topical route also avoids the systemic complications of gabapentin such as somnolence, dizziness, and peripheral edema. This study aimed to perform a narrative synthesis of studies investigating the use of topical gabapentin in the treatment of vulvodynia. The primary outcome was a change in pain score following treatment with topical gabapentin. A broad literature search was performed, which identified four studies for inclusion. The included studies reported improved pain measures following treatment; however, conclusions cannot be made due to methodological heterogeneity and inherent limitations. These include lack of control arms, small sample sizes, lack of patient randomization, and use of combination treatments. Due to the paucity of evidence, this review supports the future implementation of double-blind randomized controlled trials to further investigate the efficacy of topical gabapentin in the treatment of vulvodynia.

## Introduction

Vulvodynia is defined as vulvar pain of at least 3 months without a clear identifiable cause, typically characterized by a stinging, burning, or itching sensation (1). Vulvodynia can be classified according to location (localised or generalized), stimulus requirement (provoked or spontaneous), onset of pain (primary or secondary) and temporal pattern (intermittent or persistent) (1). The two most prevalent subtypes are provoked vestibulodynia (localised vulvodynia at the vulval vestibule in which physical contact generates pain) and generalize spontaneous vulvodynia (where pain is widespread and unprovoked) (2).

Dyspareunia describes the pain associated with sexual intercourse. Vulvodynia is the most common cause of dyspareunia in premenopausal women (3), with an estimated lifetime prevalence of 8%–16% (4). However, under-reporting of vulvodynia is common for two reasons. First, there remains poor understanding of the condition among physicians. Prior to its recent classification, vulvodynia was historically identified as a manifestation of psychological conditions (5). As such, only 10%–25% of women will be correctly diagnosed during their first visit to a gynecologist (6). Second, only approximately 60% of women will consult physicians despite experiencing symptoms (7), often because women have reported feeling stigmatized both by physicians and their families for seeking help (4).

The burden of vulvodynia extends beyond the distinctive symptom of pain. The associated pain negatively impacts sexual desire, frequency, and pleasure, and thus the intimate relationship between the woman and her partner (8). This results in detrimental effects on psychosexual health, with women disclosing feelings of chronic stress, shame, and depression (9, 10).

The precise etiology of vulvodynia is unknown, though it is widely considered to be multifactorial. Several pathophysiologic mechanisms have been proposed including genetics, inflammation, recurrent infections (e.g., candidiasis), neuropathic pain, pelvic floor muscle dysfunction, hormones, and psychosocial factors (11). The pain mechanism(s) responsible for vulvodynia follows the central sensitization paradigm as an individual experiences a hypersensitive response to pain, with resulting allodynia and hyperalgesia, in the absence of any clinically apparent pathology (12). Thus, many women with vulvodynia also have hypersensitivity at extragenital sites, associating vulvodynia with other chronic pain conditions (13, 14). Therefore, it is listed as one of the many central sensitivity syndromes, which include fibromyalgia and irritable bowel syndrome (12).

A multimodal treatment approach, tailored to the associated factors that vary patient-to-patient, is typically used to treat this complex condition. The three main management options are psychosocial interventions (cognitive behavioral therapy, sex therapy), physiotherapy to resolve pelvic floor muscle dysfunction, and pharmacologic treatment (15, 16). While some patients and physicians have claimed that medications are effective in managing the pain, the quality of scientific evidence is low (17–19). Possible agents listed in the 2021 European guidelines for the management of vulvodynia include 5% lidocaine ointment prior to penetrative sex, oral amitriptyline, and oral gabapentin (19).

Gabapentin is one of the first-line treatments recommended for neuropathic pain and is effective in several neuropathic pain conditions including postherpetic neuralgia and painful diabetic neuropathy (20, 21). Evidence for oral gabapentin in vulvodynia remains limited yet promising with several studies reporting patient satisfaction and pain relief (22–25). A limiting factor of oral therapy is the frequent systemic adverse events reported, such as somnolence, dizziness, and peripheral edema, which topical application of gabapentin may circumvent (21). Topical gabapentin is effective in animal models of neuropathic pain, although evidence in humans remains limited (26).

Following a retrospective study published in 2008, treatment with topical gabapentin was introduced more regularly into clinical practice (27). The evidence from this study was used to recommend topical gabapentin in the treatment of vulvodynia in the 2016 European guidelines, though it has been removed from the most recent guidelines for reasons unknown (28). Topical therapies, such as gabapentin formulations, are regularly used in clinical practice as they provide the benefit of reduced systemic adverse events while concurrently avoiding the interpatient variability with oral dosing regimens (29). Presently, there are no widely concurred treatment pathways for the pharmacologic management of vulvodynia due to the dearth of evidence.

Given the paucity of literature within the field, the objective of this review was to conduct a narrative synthesis of available studies that assess topical gabapentin formulations for the treatment of vulvodynia.

## Materials and methods

### Eligibility criteria

The Preferred Reporting Items for Systematic Reviews and Meta-Analyses (PRISMA) checklist was used for the reporting of this review (30). An initial study protocol was registered on PROSPERO, designed for the conduction of a systematic review. Due to the limited number of studies and their inconsistent designs, however, the authors later decided that presenting the findings in the format of a narrative synthesis would be more appropriate.

A review of randomized trials, prospective or retrospective observational studies, case reports, commentaries, and letters to editor was conducted. All studies until 10 September 2022 were included. Conference abstracts were included to limit publication bias and ensure a comprehensive overview of existing literature, as recommended by the *Cochrane Handbook for Systematic Reviews of Interventions* (31). Systematic and narrative reviews were excluded.

The population was women over the age of 18 years with vulvodynia for a minimum of 3 months. The interventions were topical formulations of gabapentin. The primary outcome was difference, if any, in pain rating scales from baseline to the last available follow-up. Any validated pain scale was accepted by authors. Other outcomes included change in sexual function, quality of life, and adverse events.

Studies evaluating patients with different types of neuropathic pain were included, provided data for the women with vulvodynia treated with topical gabapentin were explicitly reported. Exclusion criteria was assessment of other modalities of gabapentin treatment other than the topical form.

### Search

The authors conducted a literature search of the following databases: MEDLINE, EMBASE, CINAHL, the Cochrane Library, Cochrane Central Register of Controlled Trials, SCOPUS, and Web of Science. A search of EThOS and ClinicalTrials.gov was also conducted to obtain any grey literature on the topic. The reference sections of relevant original articles, reviews, and evidence-based guidelines (32) were also searched manually. The databases were searched using variations of the term “vulvodynia” and “gabapentin” combined using Boolean operators.

Covidence software was used for screening and identification of articles that met the inclusion and exclusion criteria. The search results were initially imported into Mendeley software and deduplicated, followed by importation into Covidence software and further deduplication. Titles and abstracts of the retrieved articles were then independently reviewed by four authors (ME, NC, AL, ST) and irrelevant articles excluded. Subsequently, two authors independently evaluated the full text of the remaining potential articles and determined study eligibility. Any disagreements among authors related to the eligibility criteria of the previously selected studies were discussed and a decision made by consensus.

### Data extraction

Data extraction was performed by one author and independently verified by a second author (ME, NC, AL, ST).

### Quality assessment

Quality assessment of the included studies was performed independently by two authors (ME, ST). If the authors disagreed, a third author (AL) was consulted to reach a decision through discussion. The Newcastle-Ottawa Scale was used to assess the quality of the retrospective cohort study by Aalto et al. (Appendix 1) (33, 34) The scale is an eight-item assessment tool composed of three domains: selection, comparability, and outcome of the assessed study. There is a maximum of nine stars/points across the three domains: maximum of four for selection, two for comparability and three for outcome. Each item is outlined in Appendix 1. The scoring was based on the aims of this narrative synthesis, which focussed on results for patients with vulvodynia who were treated with topical gabapentin, as opposed to the aims of the assessed study. More specifically, the comparability domain, which assessed whether certain confounders were controlled for, was scored based on whether this was achieved for the gabapentin cohort. Quality was graded as one of the following: good quality (if three or four stars in selection domain, and one or two stars in comparability domain, and two or three stars in outcome domain); fair quality (two stars in selection domain, and one or two stars in comparability domain, and two or three stars in outcome domain); poor quality (zero or one star in selection domain, or zero stars in comparability domain, or zero or one star in outcome domain) (34). The National Heart, Lung and Blood Institute (NHLBI) tool was used for the before-after studies with no control group (Appendix 2) (27, 35–37). This assessment tool contains 12 items, each evaluated as “yes”, “no”, “not applicable”, “cannot determine” or “not reported”. The responses were used to evaluate the quality of each study as either good, fair, or poor (37).

## Results

**Figure 1:**
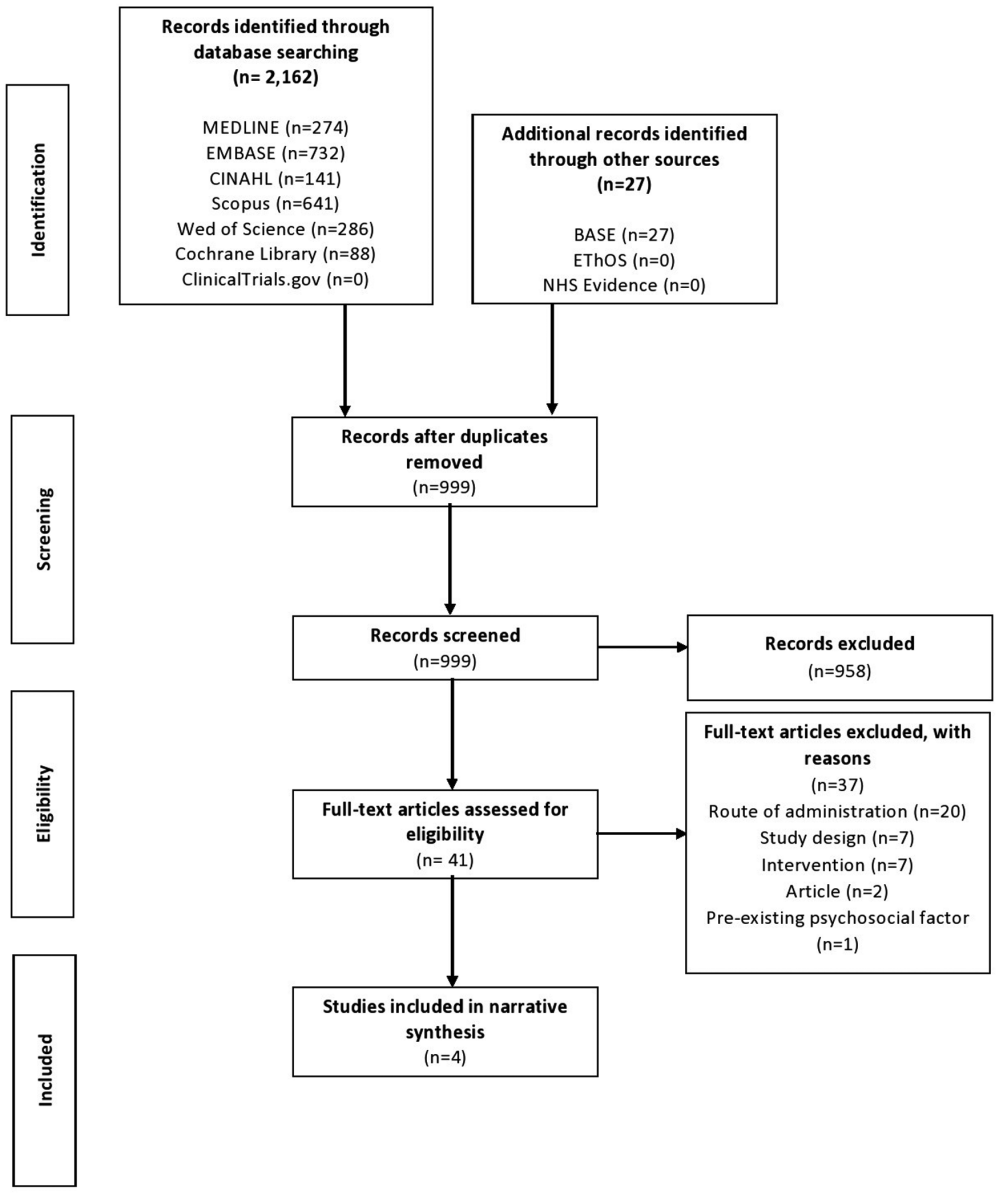
PRISMA flow diagram of search results and selection of studies for analysis.

The preliminary search yielded 2,189 results, of which 999 remained following deduplication. From the remaining studies, the selection process yielded four papers for inclusion in this review (Figure 1) (27, 33, 35, 36). All studies were observational and retrospective in nature. All studies assessed topical gabapentin 6% weight in weight (w/w), and one study also assessed gabapentin 2% and 4% w/w (27). All four studies analyzed pre-treatment assessment data vs. post-treatment assessment data.

The retrospective cohort study by Aalto et al. was deemed to be poor in quality, with the following scores for each domain: four points for selection, zero points for comparability and one point for outcome (Appendix 1) (33, 34). For the before-after studies with no control group, the quality was deemed fair, fair, and poor for the Boardman et al., Hiom et al. and Keevil et al. studies, respectively (Appendix 2) (27, 35–37).

Boardman et al. (27) conducted a retrospective study of women who presented to a vulvar clinic in the USA from 2001 to 2006 with chronic (duration 3 months or greater) vulvar pain without an identifiable underlying cause, who were treated with topical gabapentin. Three concentrations of creams, 2%, 4%, and 6%, were tested, commencing with the low dose, and titrating up if the pain-relieving effects were suboptimal. The primary outcome was an 11-point numeric rating scale (NRS), ranging 0–10 (0 indicating no pain, 10 indicating worst possible pain), both before and after treatment. Of the 210 women diagnosed with generalized or localized vulvodynia, 51 (24%) received topical gabapentin, either alone or combined with other medication(s). Many participants had tried other treatments prior to commencing gabapentin treatment (topical lidocaine (19 of 51; 37%), antifungals (17 of 51; 33%), hormonal medications (16 of 51; 31%), tricyclic antidepressants (13 of 51; 25%), and anticonvulsants (8 of 51, 16%)). When starting the topical gabapentin therapy, 19 of 51 (40%) women were also using other medications, most commonly tricyclic antidepressants and topical lidocaine. Only 35 of 51 (69%) participants completed pre- and post-treatment surveys of whom 28 (80%) reported a minimum 50% improvement in pain scores, and 10 (29%) had complete pain relief after 8 weeks of treatment. Following 8 weeks of topical gabapentin there was a decrease in mean pain score for the localized vulvodynia group from pre-treatment [7.92 ± 2.04/8 (standard deviation/median), *n* = 24] to post-treatment [2.71 ± 1.63/2.5 (standard deviation/median), *n* = 24]. Similarly, the mean pain score also decreased for the generalized vulvodynia group from pre-treatment [5.82 ± 1.72/5 (standard deviation/median), *n* = 11] to post-treatment [2.00 ± 2.32/1 (standard deviation/median), *n* = 11]. Seven of 50 (14%) women discontinued treatment, three due to local irritation and four for urinary dysfunctions such as retention, frequency, and recurrent infections. These adverse events resolved upon the termination of the treatment. None of the patients experienced adverse events typically occurring in women receiving oral gabapentin, such as dizziness and somnolence.

A single UK center, retrospective, observational study by Hiom et al. (35) investigated the safety and efficacy of topical gabapentin 6% w/w in the treatment of neuropathic pain conditions, which included five participants with vulvodynia. Gabapentin was applied three times daily and patients were followed up for assessment of pain, using the Brief Pain Inventory (an 11-point NRS, ranging 0–10, 0 indicating no pain, 10 indicating worst possible pain), quality-of-life and Chronic Pain Sleep Inventory scores over six months (38). The individual data for patients with vulvodynia displayed a general improvement in the outcome measures, though statistical analysis was not conducted. All patients in the study, including those diagnosed with vulvodynia, experienced pain improvements from 1 h after application of gabapentin (35).

Aalto et al. (33) conducted a retrospective cohort study in Finland to ascertain the effectiveness of current treatments for vulvodynia. A total of 133 patients aged greater than 18 years were included following application of the International Classification of Diseases (ICD)-10 and Friedrich's 2 criteria for vulvodynia. Questionnaires assessing vulval pain (using a NRS, ranging 0–10 with 0 indicating no pain, 10 indicating worst possible pain) and quality of life before and after treatment, and treatment satisfaction were completed by 70 (52.6%) patients. Patient demographics were also collected. The most used treatments were lidocaine gel (82.9%), physiotherapy (78.6%), counselling (74.3%), and topical gabapentin 6% (54.3%). The median NRS score of all treatments decreased from 8 to 4 following treatment, signifying a reduction in pain (*p* < 0.001) (33). There was no difference in treatment efficacy between patients with generalized or localized vulvodynia. Analysis of the NRS scores of monotherapies before and after treatment indicated no statistically significant difference. Quality of life satisfaction was associated with treatment by 67.1% of patients. Overall, the authors concluded that a combination of treatments, which included topical gabapentin, was best for addressing vulvodynia and quality of life, particularly in younger patients.

Keevil et al. (36) conducted a two-center survey in the UK to evaluate the efficacy and safety of topical gabapentin for vulvodynia (36). A total of 27 of 54 patients prescribed topical gabapentin were identified in local clinical pain and gynecology databases to participate in the survey between 2014 and 2016. An 11-point NRS was used to quantify the pain, ranging 0–10 (0 indicating no pain, 10 indicating worst possible pain), immediately and 6 months after commencement. The secondary outcomes of mood, sleep, and activity levels were recorded using a second 11-point scale (−5 to 5). Overall satisfaction with gabapentin use was also recorded. The 14 patients who responded to treatment experienced a mean reduction in pain of 66% immediately with 49% women continuing to experience pain relief after 6 months. Sleep, mood, and activity were improved in five, ten, and eight patients but decreased in one, two, and one patient(s), respectively. Overall, the authors concluded that this survey supports the use of topical gabapentin for vulvodynia, reducing pain and improving quality of life indicators.

Table 1 summarizes the study designs, sample sizes, treatment, and results from the four included studies (Table 1).

**Table 1:** Summary of the characteristics, findings and quality of the included studies.

Reference	Study design	Sample size	Intervention	Outcome	Results	Side-effects	Quality
Aalto et al. (33)	Retrospective cohort study	38	6% gabapentin gel	11-point NRS pre- and post-treatment.	Pre-treatment pain: 8 (IQR: 8–9), reduced to 5 (IQR: 3–7) after treatment. *p* Value was non-significant.	Not reported	Poor
Boardman et al. (27)	Retrospective before-after study	51 total, 35 included in analysis	2%, 4% or 6% gabapentin gel	11-point NRS pre- and post-treatment.	Reduction in pain from 7.26 to 2.49 after minimum of 8 weeks of treatment	3 patients experienced local irritation. 4 had urinary symptoms.	Fair
Hiom et al. (35)	Retrospective before-after study	5	6% gabapentin gel	11-point NRS measured every 4 weeks during treatment.	Data presented as a graph, no statistical analysis of the data	No patients with vulvodynia had adverse effects.	Fair
Keevil et al. (36)	Retrospective before-after observational study	54 total, 27 included in analysis	Gabapentin gel—strength not reported	11-point NRS immediately after commencing treatment and after 6 months of treatment	Improved pain reported by 14 patients. In those who responded to treatment: 66% experienced mean reduction in pain immediately and 49% after 6 months.	2 patients experienced local irritation.	Poor

NRS, numeric rating scale.

## Discussion

The studies in this review suggest a correlation between the use of topical gabapentin and improvement in pain scores for patients with vulvodynia. Although many patients used other pain treatments in conjunction with topical gabapentin, the Boardman et al. study suggests improvement in pain scores and a low number of adverse events. The other three studies by Aalto, Hiom and Keevil also show a similar correlation in results. It must be stated, however, that solid conclusions about the efficacy and safety of topical gabapentin in vulvodynia cannot be drawn at present due to the methodological limitations of each study.

The internal validity of the Boardman et al. study was limited as a larger number of patients were receiving other concurrent pharmaceutical treatments, compounded by the lack of a control arm (27). The study was also deemed to be fair in quality using the NHLBI tool (Appendix 2). Additionally, the effect of the different gabapentin concentrations (2%, 4% and 6%) were not analyzed due to the small sample size. The study by Hiom et al., which was found to be fair in quality (Appendix 2), had only four patients, which reduced the study's power (35). Data were presented graphically and showed a trend suggesting improvement in pain although statistical analysis was not conducted. Additionally, the article was a letter to the editor, thus the methodology was not clear. The aetiology for chronic pain was not clearly defined whilst the results were not statistically analyzed before being presented in the form of a graph. The Aalto et al. study, deemed poor in quality using the Newcastle-Ottawa Scale (Appendix 1), was limited by its questionnaire-based design with a low response rate (52.6%) increasing risk of selection bias. Patients were asked to recall their pain retrospectively with a range of one year to 11 years between treatment administration and when the questionnaire was answered, further increasing risk of bias. Topical gabapentin was not given in isolation as patients received a combination of treatments with no indication as to which combinations were used, thus the efficacy of the topical gabapentin alone cannot be delineated (33). Whilst the study data by Keevil et al. suggests a positive trend in pain improvement, there was neither a control arm nor a clear explanation in the methods to whether the study was standardized for all patients (36). No demographic information was included, so it is difficult to evaluate the generalizability of the study. The study was also found to be poor in quality using the NHLBI tool (Appendix 2). The patient population was non-randomized and of 54 potential participants, investigators were only able to contact 27 of them leading to potential selection bias. Furthermore, this study was presented as a poster and therefore did not undergo formal peer-review (36).

Although the included studies have inherent weaknesses, they are the first to investigate the efficacy and safety profile of topical preparations of gabapentin in the treatment of chronic pain conditions. Indeed, the positive findings have been corroborated by studies involving animal pain models. Application of topical gabapentin (10% gel) significantly reduced allodynia and vulvodynia in a streptozotocin-induced diabetic neuropathic rat model (39). An analgesic effect was also observed in a rabbit model in which ocular pain was ameliorated following application of gabapentin eye drops (40). Furthermore, gabapentin 10% gel attenuated neuropathic allodynia and heat-hypoalgesia in a cisplatin rat model of chemotherapy-induced peripheral neuropathy (41). Topical gabapentin 10% gel also significantly allayed hyperalgesia and allodynia in a rat chronic sciatic nerve constriction injury neuropathic pain model (26). These studies involving animals provide evidence of the potential of topical gabapentin use in the treatment of neuropathic pain, although it must be emphasized that the models do not directly translate to clinical settings.

Other topical treatments have been explored for the treatment of vulvodynia, owing to their localised effect and fewer systemic adverse events. Kim et al. demonstrated that the use of combined topical 0.3% meloxicam and 5% lidocaine for one week resulted in amelioration of pain symptoms for 75% of participants (*n* = 8). The sample size was small, however, and there was difficulty in ascertaining the extent to which lidocaine and meloxicam alone influenced the findings (42). The benefit of topical lidocaine was further supported by a prospective before-and-after study, in which 5% lidocaine ointment led to 76% of participants (*n* = 61) reporting the ability to experience intercourse after the treatment course compared to 36% prior to treatment (*p* = 0.002). A statistically significant decrease in intercourse-related pain score (*p* < 0.001) and daily pain score (*p* = 0.004) was also demonstrated (43). Another study reported that topical lidocaine resulted in significant increase in pain thresholds, measured using a vulvar-algesiometer, and a small but significant reduction in pain using the visual analogue scale at 12 months compared to baseline (*p* = 0.007 and *p* = 0.04, respectively) (44). A multicentre, randomised trial showed data for the overnight use of 5% lidocaine ointment for 10 weeks. Patients had a statistically significant reduction in pain after lidocaine treatment compared to baseline (*p* < 0.001), though multimodal physical therapy, the other treatment investigated in this study, was more effective in reducing pain (45). In a randomised controlled trial, the use of topical lidocaine displayed pain reduction though this was insignificant compared to placebo (46). Adverse events were exhibited in some studies. Firstly, Kim et al. reported localised adverse events of burning and stinging, though the study does corroborate that topical therapies avoid systemic adverse events (42). Secondly, Danielsson et al. found the only reported adverse event was localised stinging pain whilst systemic adverse events were not reported (44). Lastly, Morin et al. outlined that one patient dropped out from the study due to a dermatitis reaction to the lidocaine ointment. A further 15 women reported minor sensation of irritation or burning, though not severe enough to warrant discontinuation of treatment (45). A study in which 2% amitriptyline cream was used in the management of dyspareunia due to provoked vestibulodynia found that 56% (*n* = 84) of patients displayed improvement in pain score and that 10% (*n* = 15) were completely pain free and considered themselves as cured (47). Poterucha et al. investigated the use of a topical amitriptyline-ketamine combination for the treatment of pelvic pain. Of the 13 patients included, 7 displayed relief from use of the topical agent, though it is unclear which of these patients had pain of vulvar aetiology (*n* = 4). Only one patient reported irritation when lidocaine was also added to the amitriptyline-ketamine combination (48). Another study demonstrated that amitriptyline 2%/baclofen 2% cream in the treatment of provoked vestibulodynia resulted in 71% of patients reporting at least moderate improvement in symptoms, and a statistically significant decrease in pain during intercourse and its effect on social activities. Eleven (29%) of women experienced localised burning, of which three discontinued treatment because of this adverse event (49).

There is a paucity of evidence for the use of topical gabapentin in other neuropathic pain conditions. A case report described the alleviation of pain caused by trigeminal neuralgia with the use of 6% gabapentin cream, with the effects being sustained for up to 6 months of continuous therapy (50). A randomised controlled trial investigating use of compounded cream of ketamine, gabapentin, clonidine and lidocaine in neuropathic pain showed reduced pain after 1 month, however there was no significant difference compared to placebo (51). This study included patients with many types of neuropathic pain so it remains possible specific aetiologies may have benefited from this compounded cream. Topical gabapentin has been investigated in various other conditions. In CKD-associated pruritus, 6% gabapentin cream significantly reduced pruritus VAS score after 2 weeks of therapy. The mean reduction in VAS pruritus scores from base line to week 2 were significantly greater in the treatment group compared to control, showing potential efficacy for short term treatment in this condition (52). Lastly, Saki et al. conducted a randomised control trial investigating use of 10% gabapentin cream in treating epidermolysis bullosa pruritus. This trial showed no significant difference between gabapentin and control in treating erythema and pruritus after 6 weeks of therapy (53).

## Conclusion

This narrative review collated the current evidence to ascertain the effect of topical gabapentin for the treatment of patients with vulvodynia. Whilst there is a favorable trend across the included studies, the degree of heterogeneity across their methodologies means the effects of topical gabapentin to improve pain in vulvodynia cannot be discerned. Moreover, the lack of a control arm in the studies fails to eliminate the possible placebo effect of the gabapentin formulations. Indeed, the efficacy of topical gabapentin has been displayed through animal models investigating pain, which support continued studies in this area. Therefore, with shared decision making, after review of the suggestion of pain improvement, but with absence of robust evidence, topical gabapentin may be used either as adjunctive or alternative treatment for vulvodynia, posing an advantage over oral formulation as it is devoid of systemic adverse events. This review can lend weight to support the conduction of double-blinded randomized controlled trials. These studies must be robust and aim to investigate various aspects of topical gabapentin use: (1) effects of different doses; (2) effects of different daily regimens; (3) primary outcome measure using standardized assessment of pain severity and secondary measures including but not limited to quality-of-life; (4) effects both stand-alone and as an adjunct; (5) adverse events profile.

## Appendix 1 Newcastle-ottawa scale.

**Table:**

Author year	Selection				Comparability	Outcome			Quality
	S1	S2	S3	S4	C1	O1	O2	O3	
Aalto et al. (33)	Somewhat representative of the average vulvodynia patient in the community*	Drawn from the same community as the exposed cohort*	Secure record (e.g., surgical records)*	Yes*	Cohorts are not comparable on the basis of the design or analysis controlled for confounders	Self-report	Yes*	Follow up rate less than 80% and no description of those lost	Poor

S1, representativeness of the exposed cohort; S2, selection of the non-exposed cohort; S3, ascertainment of exposure; S4, demonstration that outcome of interest was not present at start of study; C1, comparability of cohorts on the basis of the design or analysis; O1, assessment of outcome; O2, was follow-up long enough for outcomes to occur; O3, adequacy of follow-up of cohorts.

* Signifies a response that attained a star/point.

## Appendix 2 NHLBI quality assessment tool for before-after (pre-post) studies with no control group.

**Table:**

Author year	Q1	Q2	Q3	Q4	Q5	Q6	Q7	Q8	Q9	Q10	Q11	Q12	Quality
Boardman et al. (27)	Y	Y	Y	Y	NR	Y	Y	N	N	Y	N	NA	Fair
Hiom et al. (Gabagel) (35)	Y	N	NR	NR	CD	NR	Y	NR	Y	Y	Y	NA	Fair
Keevil et al. (36)	Y	Y	NR	N	CD	N	N	NR	NR	N	N	NA	Poor

Q1, was the study question or objective clearly stated?; Q2, were eligibility/selection criteria for the study population prespecified and clearly described?; Q3, were the participants in the study representative of those who would be eligible for the test/service/intervention in the general or clinical population of interest?; Q4, were all eligible participants who met the prespecified entry criteria enrolled?; Q5, was the sample size sufficiently large to provide confidence in the findings?; Q6, was the test/service/intervention clearly described and delivered consistently across the study population?; Q7, were the outcome measures prespecified, clearly defined, valid, reliable, and assessed consistently across all study participants?; Q8, were the people assessing the outcomes blinded to the participants' exposures/interventions?; Q9, was the loss to follow-up after baseline 20% or less?; Were those lost to follow-up accounted for in the analysis?; Q10, did the statistical methods examine changes in outcome measures from before to after the intervention?; Were statistical tests done that provided *p* values for the pre-to-post changes?; Q11, were outcome measures of interest taken multiple times before the intervention and multiple times after the intervention (i.e., did they use an interrupted time-series design)?; Q12, if the intervention was conducted at a group level (e.g., a whole hospital, a community, etc.) did the statistical analysis consider the use of individual-level data to determine effects at the group level?; Y, yes; N, no; CD, cannot determine; NA, not applicable; NR, not reported.
